# Valorization of Food Industry By-Products for Sustainable Functional Food Production: Recent Advances and Future Perspectives

**DOI:** 10.3390/foods15122116

**Published:** 2026-06-12

**Authors:** Lina Merino, Manuel Teijeiro, Juan Manuel Castagnini, Albert Sebastià, Francisco J. Martí-Quijal, Paula Bucci

**Affiliations:** 1 Laboratory for Research on New Foods and Nutrition (LINAN), National University of Hurlingham (UNAHUR), Tte. Origone 151, Hurlingham 1688, Argentina; lina.merino@unahur.edu.ar (L.M.); paulabucci1@gmail.com (P.B.); 2 National Food Institute, Estados Unidos 25, Buenos Aires 1101, Argentina; manuteije@gmail.com; 3 Research Group in Innovative Technologies for Sustainable Food (ALISOST), Department of Preventive Medicine and Public Health, Food Science, Toxicology and Forensic Medicine, Faculty of Pharmacy and Food Science, Universitat de València, Avda, Vicent Andrés Estellés, 22, Burjassot, 46100 Valencia, Spain; juan.castagnini@uv.es; 4 Institute of Sustainable Processes, University of Valladolid, Doctor Mergelina s/n, 47011 Valladolid, Spain; 5 Foundation for Research and Development in Transport and Energy (CIDAUT), Parque Tecnológico de Boecillo, 47051 Valladolid, Spain

**Keywords:** circular economy, food by-products, functional foods, sustainability, food innovation, waste

## Abstract

Food industry by-products represent an abundant and underexploited source of bioactive compounds, dietary fibers and proteins with significant potential for functional food development. Recent studies estimate that up to 30 to 50% of processed raw materials are discarded as by-products, while food waste contributes approximately 8–10% of global greenhouse gas emissions, equivalent to nearly 3.3 billion tons of CO_2_ annually. This review critically evaluates advances (2015–2026) in the valorization of food industry by-products, with a focus on technological efficiency, health-related evidence, and environmental impact. Specifically, it addresses the following research question: to what extent do current valorization strategies provide measurable technological, nutritional, and environmental advantages over conventional food production systems? Emerging extraction technologies including ultrasound- and microwave-assisted extraction (20–40 kHz, 30–60 °C), supercritical fluid extraction (200–350 bar, 35–60 °C), enzymatic hydrolysis, and fermentation demonstrated improvements in extraction yields (up to 20–50% increases compared to conventional methods) and higher purity in the recovered compounds. These approaches enable the isolation of compounds such as pectins from citrus peels, polyphenols from grape pomace, galacto-oligosaccharides from dairy whey, and collagen from fish by-products. From an environmental perspective, valorization strategies can reduce waste disposal and associated emissions by up to 30%, depending on the scale and type of by-product processing. Furthermore, these approaches contribute directly to circular economy models and support multiple Sustainable Development Goals, particularly SDG 12 (responsible consumption and production) and SDG 13 (climate action). However, challenges remain, including variability in raw material composition, scalability limitations, and the limited availability of high-quality clinical evidence supporting health benefits. By integrating nutritional potential, technological feasibility, and sustainability indicators, this review provides a comprehensive and critical assessment of the current state of by-product valorization and identifies key gaps for future research.

## 1. Introduction

The global food system is currently under increasing pressure due to population growth, resource constraints, and the environmental impact of food production demanding innovative solutions. Among these challenges, food waste and the inefficiency of resource utilization stand out as critical issues. According to the Food and Agriculture Organization (FAO) and the United Nations Environment Programme (UNEP), in 2022, approximately 1.05 billion tons of food was wasted annually across the world (2024), representing 19% of the total food available to consumers. Food waste not only exacerbates environmental degradation, accounting for between 8% and 10% of global greenhouse gas emissions, but it also generates economic losses and underutilized nutritional potential. Simultaneously, according to United Nations population prospects, the global population is projected to reach 9.7 billion by 2050, further escalating the demand for sustainable and nutritious food sources. By-products and residual streams generated in the food industry include shells, seeds, peels, pulp, whey, bones, skin, cartilage, wastewater with organic load, and off-specification batches. Depending on the sector and processing chain, a substantial proportion of these materials remains rich in recoverable proteins, fibers, lipids, and bioactive compounds, making them suitable for valorization into food ingredients, feed, bioenergy, or biomaterials. In this context, the valorization of food industry by-products has emerged as a promising strategy to address these concerns by transforming waste streams into valuable nutritional resources [1,2,3].

The concept of functional foods, which dates back to Japan in the 1980s and was formalized under the Foods for Specified Health Uses (FOSHU) framework, has further propelled this innovation. Functional foods are defined as those fortified with specific components that provide health benefits beyond basic nutrition [4]. The rising demand for health-promoting foods, coupled with increasing environmental awareness, has intensified efforts to incorporate by-products from the food industry into functional and sustainable food solutions. The estimated functional foods market size in 2025 was over USD 300 billion, almost doubling its 2018 figures, mainly due to this consumer awareness, with an annual growth of 8.5% For instance, the circular economy model, which prioritizes minimizing waste and maximizing resource efficiency, offers a viable alternative to the traditional linear production model of extraction, production, and disposal [5]. This is especially relevant considering that approximately one-third of all food produced for human consumption—around 1.3 billion tons annually—is lost or wasted.

The global market for functional foods, nutraceuticals, and bioactive ingredients derived from sustainable and natural sources has experienced significant growth in recent years, driven by increasing consumer demand for health-promoting and environmentally sustainable products. Recent market analyses estimated annual growth rates exceeding 7–9% for functional bioactive ingredients and nutraceutical products, while the global nutraceutical market is projected to surpass USD 600 billion within the next decade [6,7,8]. This expansion has been particularly notable within the clean-label and sustainable food sectors, where consumers increasingly associate natural and upcycled ingredients with health, environmental responsibility, and product quality. In this context, food-industry by-products are increasingly being recognized as economically valuable raw materials for the development of sustainable functional foods, bio-based ingredients, and circular economy strategies [9].

By-products from the food industry, ranging from fruit peels and seeds to dairy whey and fish trimmings, have historically been relegated to low-value uses such as animal feed or composting. However, emerging research highlights their potential as sources of bioactive compounds, dietary fibers, proteins, and other valuable ingredients. For example, citrus peels, rich in flavonoids and pectin, can be transformed into functional ingredients with antioxidant and gut health benefits [5]. Similarly, spent grains from brewing, often discarded as waste, contain high levels of proteins and fibers suitable for developing functional food products aimed at improving health outcomes [10].

The adoption of these practices aligns with global policy frameworks, such as the European Green Deal and the United Nations Sustainable Development Goals (SDGs), which emphasize sustainable production and waste reduction [1,2,3,11]. By utilizing by-products, food producers can not only mitigate environmental impacts but also enhance economic resilience by generating additional revenue streams. Nevertheless, significant barriers remain, including variability in by-product composition, the cost of extraction technologies, and the need for consumer acceptance of foods derived from waste streams [12].

This review aims to explore these opportunities, examining how innovations in by-product utilization can contribute to sustainable and nutritious food systems. It highlights the transformative potential of these approaches, while also addressing the challenges that must be overcome for their widespread adoption in the global food industry. This approach should also be considered during the design of legislation aimed at regulating the use of by-products and public policy for the transition from a linear production model to a circular economy model.

## 2. Materials and Methods

We conducted a structured narrative review of scientific literature published between 2015 and 2024 on the valorization of food industry by-products for the development of functional foods and their potential impacts on human health. The search was run in Scopus, Web of Science (Core Collection), and Google Scholar. The search strategy combined terms for food-industry by-products/side-streams (e.g., “by-product,” “side-stream,” “residue,” “pomace,” “bagasse,” “bran,” “spent grain,” “whey,” “fish skin/scales,” “meat trimmings”), functional foods/bioactive ingredients (e.g., “functional food,” “nutraceutical,” “bioactive,” “fortification,” “dietary fiber,” “polyphenol,” “peptide,” “omega-3,” “prebiotic”), and health-related contexts across the plant, dairy, meat, and fish sectors, including human, *in vivo*, and *in vitro* models with human relevance.

**Figure 1:**
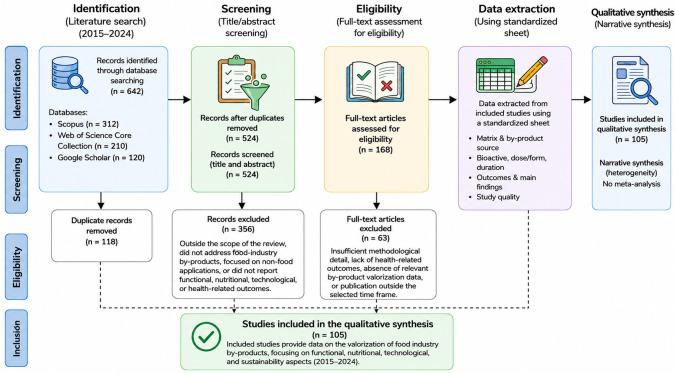
Methods and structure of narrative review.

Inclusion criteria: (i) peer-reviewed studies developing foods or ingredients derived from plant, dairy, meat, or fish by-products; and (ii) reporting health-related outcomes (human trials, *in vivo*, or *in vitro* with human relevance). Exclusion criteria: non-food applications (e.g., materials, energy, feed), non-eligible document types (editorials, letters), abstracts without full text, studies outside 2015–2026, or studies lacking health outcomes. Title/abstract screening was followed by full-text assessment. Data extraction used a standardized sheet (food matrix, by-product source, derived compound, dose/form, duration, outcomes, main findings, study quality). Owing to heterogeneity in designs, matrices, and endpoints, the findings were synthesized qualitatively; no quantitative meta-analysis was performed (Figure 1).

The literature selection process followed PRISMA guidelines for transparent and reproducible reporting. In total, 642 records were initially identified through database searching, including Scopus, Web of Science Core Collection, and Google Scholar. After the removal of 118 duplicate records, 524 records were screened by title and abstract. Of these, 356 records were excluded because they were outside the scope of the review. Subsequently, 168 full-text articles were assessed for eligibility, resulting in 105 studies included in the final qualitative synthesis.

## 3. Development of New Foods from By-Products: Main By-Products and Their Nutritional Potential—Nutritional Composition

### 3.1. Vegetable By-Products

The management of fruit and vegetable waste and by-products remains a critical global challenge. Despite increasing efforts to reduce food waste, large quantities of materials continue to be discarded from primary production and throughout various stages of the food production value chain. Several authors and international organizations have provided significant data on this issue. According to Baysal and Ülkü [13], approximately 33% of annual food production is either lost or wasted. Similarly, FAO [14] estimates that this figure amounts to roughly 1.6 billion tons annually, contributing to about 3.3 billion tons of CO_2_ emissions.

Trigo et al. [15] note that a considerable portion of waste is generated during food processing, where secondary by-products from raw material transformation are frequently discarded. Rifna et al. [16] emphasize that the horticultural sector generates higher quantities of residual by-products than other food industries, with peels accounting for 25–30% of total waste, alongside kernels, pods, capsules, mashed pulp, and other remnants.

Recent estimates published between 2023 and 2024 indicate that global food waste generation exceeds 2.5 billion metric tons annually across the food supply chain, reflecting a substantial increase compared to previous decades. According to the United Nations Environment Program (UNEP) Food Waste Index Report 2024, approximately 1.05 billion tons of food waste was generated at the retail, food service, and household levels in 2022 alone, representing nearly 19% of food available to consumers. In parallel, the Food and Agriculture Organization [1,2,3] (FAO, 2023) estimates that approximately 13–14% of food produced globally is lost between the harvest and retail stages before reaching consumers. In the United States, recent analyses reported that between 30% and 40% of the national food supply is wasted annually, while household losses associated with fruit spoilage alone may exceed USD 500 per consumer per year.

Fruit waste occurs both on farms, due to pests and diseases, and throughout post-harvest stages such as storage and transportation—often due to inefficient practices [17]. Additionally, large amounts are discarded in supermarkets and households. In Europe, data show that fruit and vegetable waste accounts for over 50% of total food waste [18]. Zhu et al. [19] highlight the significant economic losses experienced by the fruit and vegetable sector due to millions of tons of waste. These residues, although often treated as waste, contain bioactive compounds with functional properties, such as antioxidant and antibacterial effects.

During the industrial food processing of widely consumed vegetables including potatoes, tomatoes, and carrots, significant quantities of waste are produced. For example, pectin-rich fractions obtained from carrot residues exhibit strong antioxidant activity due to their content of α- and β-carotenes, lutein, and tocopherols. Pectin is also one of the main compounds extracted from apple and citrus fruit waste and is highly valued for its functional properties as a thickener, gelling agent, and food stabilizer [20].

Beyond food applications, fruit and vegetable residues are also being explored as raw materials for biofuel production using biorefinery technologies such as fermentation, pyrolysis, gasification, anaerobic digestion, and hydrothermal carbonization. Advanced extraction technologies such as ultrasound-assisted extraction (UAE), microwave-assisted extraction (MAE), supercritical fluid extraction (SFE), and high hydrostatic pressure (HHP) have significantly improved the recovery efficiency of bioactive compounds from food by-products compared to conventional extraction methods. Recent studies reported that UAE and MAE increased the extraction yield of polyphenols, carotenoids, and pectins by approximately 20–50%, while simultaneously reducing the extraction time and solvent consumption by up to 60% and 40%, respectively [16,21]. Similarly, SFE using CO_2_ under pressures between 200 and 350 bar demonstrated higher selectivity and purity in the recovery of lipids and phenolic compounds, while considerably decreasing the use of organic solvents and reducing the environmental burden associated with conventional extraction systems [21].

From an environmental perspective, the implementation of green extraction technologies has been associated with substantial reductions in energy consumption and greenhouse gas emissions. Life cycle assessment studies suggested that optimized valorization strategies may reduce CO_2_-equivalent emissions by approximately 15–30%, depending on the by-product matrix and extraction process employed [22]. Furthermore, the reutilization of food processing residues contributes to reducing landfill disposal and methane emissions, reinforcing the role of these technologies within circular economy models and sustainable food production systems [22]. Together, these strategies not only significantly reduce organic waste but also pave the way for a more sustainable food production system, in alignment with the United Nations Sustainable Development Goals.

### 3.2. Dairy Industry By-Products

The processing of animal-based products, particularly within the milk and meat industries, generates substantial volumes of waste. The dairy industry generates substantial volumes of by-products, particularly whey, which is the main residual stream derived from cheese manufacturing. It is estimated that approximately 9 L of whey is produced for every kilogram of cheese manufactured, resulting in more than 180–200 million tons of whey generated annually worldwide [23,24]. Due to its high organic load, untreated whey represents an important environmental concern, with biological oxygen demand (BOD) values ranging from 30 to 50 g/L and chemical oxygen demand (COD) values reaching up to 80 g/L [25]. However, whey also contains valuable nutrients, including lactose, whey proteins, minerals, and bioactive peptides, making it a promising substrate for the development of functional foods and nutraceutical ingredients.

Whey proteins can be enzymatically hydrolyzed to release bioactive peptides with antioxidant and antihypertensive properties [26]. After undergoing microfiltration, the protein-rich retentate is recovered, while the carbohydrate-rich permeate, primarily composed of lactose and oligosaccharides, can be further valorized. One notable application is the production of galacto-oligosaccharides (GOS) with prebiotic activity through the transgalactosylation of lactose using β-galactosidase [27]. These compounds are known to promote the growth of beneficial gut microbiota, such as Bifidobacteria, and they exhibit anti-inflammatory effects and the inhibition of pathogenic bacterial adhesion to intestinal cells. Whey fermentation has also been demonstrated as an alternative valorization pathway for this by-product of the dairy industry. Pescuma and colleagues [23] reviewed the multiple uses of whey as a sustainable raw material for the production of individual compounds, foods, and beverages by microbial fermentation. It is interesting to note that whey fermented with microorganisms from milk kefir has shown antimicrobial and immunomodulatory activity [28]. The high protein quality of whey fermented with water kefir grains has also been studied [29].

In addition to traditional whey, protein concentrates such as α-lactalbumin and β-lactoglobulin have attracted attention for their use in nutritional and bodybuilding supplements, owing to their digestibility and amino acid profiles [26]. Moreover, unflavored whey protein concentrates are increasingly incorporated into functional food formulations.

Emerging studies also highlight the value of goat milk whey hydrolysates, which are shown to contain antifungal peptides that may be used as natural preservatives in bread production [30]. This application not only extends shelf life but also supports cleaner label initiatives in the baking industry.

Another underutilized yet highly valuable dairy by-product is colostrum, the initial milk produced by mammals’ post-parturition. Although its presence in commercial milk is often undesirable due to sensitivity to heat and flavor issues, colostrum is rich in immunologically active proteins, with immunoglobulins accounting for approximately 50% of total protein content [20]. Lactoferrin, a key glycoprotein found in colostrum, possesses notable antioxidant, antimicrobial, anti-inflammatory, and neuroprotective properties [21]. Through microfiltration, colostrum permeate can also be processed to isolate bioactive milk oligosaccharides with significant health-promoting effects [26].

While most research has focused on widely consumed milks such as those from cows, buffalo, goats, and sheep, there remains considerable untapped potential in the by-products of less commonly used milk sources such as camel and donkey. These could represent novel opportunities for functional food innovation and waste valorization in the future (Table 1).

### 3.3. Valorization of Marine By-Products in the Food and Cosmetic Industries

The marine product processing industry generates significant quantities of waste, including fish skin, bones, viscera, and shrimp shells, which offer substantial potential for valorization. These by-products can be transformed into high-value ingredients such as fish oil, rich in long-chain omega-3 polyunsaturated fatty acids (PUFAs), which are known for their health benefits and can be used to enrich both dairy and non-dairy food products [31]. In addition, fish proteins, rather than cholesterol, play a central role in NCDs and are a valuable source of bioactive peptides that are easily digestible and possess functional properties, making them suitable for use in soups, bakery goods, and infant formulas [32,33].

Furthermore, effluents generated during the process of cooking snow crab in seafood processing plants, typically considered as waste, can be concentrated and repurposed as natural food flavorings, providing an eco-friendly alternative for the food industry [34]. This approach not only adds value to waste materials but also reduces environmental impact.

Beyond food applications, seafood waste is also being explored for its utility in the cosmetics and pharmaceutical industries. Chitosan, a biopolymer derived from shrimp and crab shells, has shown wide-ranging applications due to its antimicrobial, antitumor, and film-forming properties. It is already used in cosmetic formulations and has potential in drug delivery systems. Enzymes, gelatin, and other proteins isolated from seafood waste also display bioactive properties that further expand their applicability across various industries.

Shrimp processing alone contributes a significant share of marine waste, with shrimp heads and shells accounting for 71.4% and 28.6% of the waste, respectively [35]. These wastes are rich in proteins, lipids, and the antioxidant astaxanthin pigment, making them ideal candidates for extraction and use in value-added seafood products [23]. Notably, shrimp head protein hydrolysates (SHPH), prepared from various species such as *Pandalus eous*, *Metapenaeus endeavouri*, and *Penaeus monodon*, have shown high protein content (90–91%), with amino acids accounting for 71–84% of the total composition, while containing minimal fat (0.01–0.02%) [36].

These hydrolysates provide functional advantages, such as preventing the denaturation of myofibrillar proteins during dehydration processes by stabilizing water molecules, which is particularly useful in the production of intermediate moisture foods. This functional role further highlights SHPH as a natural food additive with both nutritional and preservative benefits.

In recent years, there has been a focus on the use of innovative technologies to add value to marine by-products. For example, in 2021, Tremblay et al. [37] explored various non-thermal processing technologies (such as pulsed electric field, high hydrostatic pressure, membrane technology and ultrasound-assisted extraction), finding that they not only improved the extraction yield of value-added components but also reduced processing times and solvent consumption.

Overall, the valorization of marine by-products represents a promising opportunity to advance circular economy principles in the seafood sector, fostering environmental sustainability, economic efficiency, and cross-industry innovation. Recent studies indicate that 30 to 70% of total processed marine biomass may become by-products or residual streams, including heads, shells, skin, bones, and viscera, many of which remain rich in proteins, collagen, lipids, and bioactive compounds [33,38]. The recovery and reutilization of these materials can significantly reduce organic waste generation while creating additional economic value through the production of functional ingredients, nutraceuticals, and biomaterials [9,39].

Globally, the seafood processing industry generates millions of tons of by-products annually, representing approximately 30–70% of the total processed biomass depending on the species and processing method. Shrimp processing alone can generate up to 50–60% waste, mainly composed of shells and heads that are rich in proteins, lipids, chitin, and astaxanthin. Similarly, fish filleting operations may generate between 20% and 80% residual biomass, including skin, bones, and viscera, which can be valorized into collagen, bioactive peptides, and omega-3-rich oils.

## 4. Health and Nutrition

Functional foods derived from food industry by-products have attracted increasing attention due to their potential role in promoting health and preventing non-communicable diseases (NCDs). However, the strength of the available scientific evidence varies considerably. Most findings are based on *in vitro* assays and animal models, which highlight mechanisms such as antioxidant and anti-inflammatory activity, improvement of the gut microbiota balance, and the production of short-chain fatty acids (SCFAs) from fiber-rich residues. Some human studies provide preliminary support. For example, Brazil nut consumption improved lipid markers in diabetic and healthy individuals, while date seed supplementation reduced oxidative stress and inflammation. Whey-derived peptides and fermented by-products have also been associated with antihypertensive or immunomodulatory effects, although mainly in small-scale interventions. Despite these promising findings, large, well-controlled clinical studies remain limited, and consumer acceptance of foods derived from by-products continues to represent an important challenge.

Overall, current evidence suggests that valorized by-products may contribute to reducing disease risk and improving nutritional quality, but additional translational research is essential to confirm efficacy, safety, dose–response relationships, and long-term applicability. Furthermore, although some meta-analytical evidence supports the beneficial effects of specific bioactive compounds such as polyphenols and bioactive peptides, further standardized clinical investigations are still necessary to establish regulatory applicability and industrial scalability of these sustainable functional ingredients.

### 4.1. Functional Foods Against Non-Communicable Diseases

Researchers at the intersection of sustainable food systems and functional foods face a two-fold challenge: making food systems more sustainable and ensuring that foods combat non-communicable diseases (NCDs) such as cardiovascular diseases (CVDs), diabetes, and obesity. Inflammation is the primary cause of NCDs [40]. Addressing this through dietary interventions is essential.

Although cholesterol has often been the main focus of such research, recent evidence suggests that inflammation, rather than cholesterol, plays a central role in NCDs [40]. The COVID-19 pandemic further emphasized inflammation’s central role, especially among individuals with chronic diseases [41].

Several studies highlight functional foods with anti-inflammatory properties. Brazil nuts improve lipid markers in both healthy individuals and diabetics [42]. Date seed consumption reduces oxidative stress and inflammation [43]. Spirulina sauce improves liver function and lipid profiles [44]. Additionally, some dairy by-products have been successfully tested *in vitro* for anti-inflammatory and antihypertensive properties [45]. Moreover, in recent years, anti-inflammatory and immunomodulatory properties have been related not only to probiotics, but also to inactivated microbial cells (non-viable) or “postbiotics” as reviewed by Siciliano et al. [44] in 2021.

### 4.2. Functional Foods, the Gut Microbiome, and Side Stream Valorization

**Figure 2:**
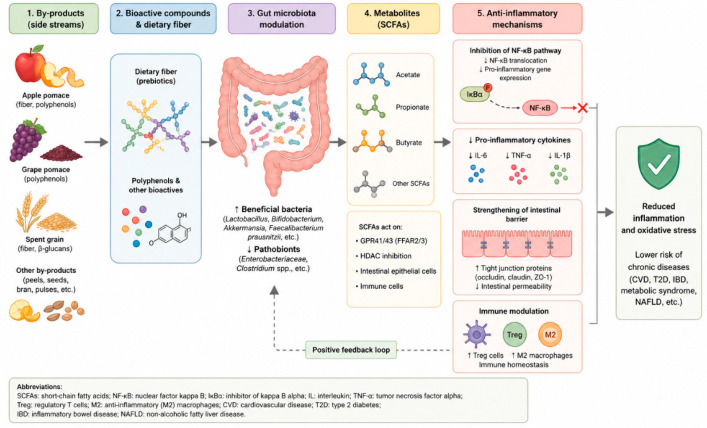
Mechanistic anti-inflammatory pathway of bioactive compounds and dietary fiber from food industry by-products.

Future research should also focus on the gut microbiome’s role in inflammation regulation. High-income countries show declining fiber intake and rising autoimmune diseases. Studying dietary fiber and microbiota interactions is crucial [46]. The gut microbiome regulates inflammation and supports health [47]. In this sense, side streams rich in dietary fiber, such as fruit pomaces, can be fermented to produce short-chain fatty acids (SCFAs) that reduce inflammation [48]. In addition, emerging technologies can extract bioactive nutrients from pomaces [49] (Figure 2).

Research on olive and apple pomaces has demonstrated anti-inflammatory effects and potential applications in animal/fish feed [50]. In addition, carbohydrate-based prebiotics present in these by-products contribute to gut health modulation [51]. These functional interactions can be further optimized using machine learning approaches [52]. Although further studies are still needed to unleash their full potential, the use of artificial intelligence and machine learning allows for the optimization of data analysis and predictions of the impact of consuming these functional foods, opening up a new range of AI-based food solutions [53,54].

### 4.3. Sustainably Produced Nutraceuticals: Examples and Trends

To counteract environmental degradation from food production, new research focuses on sustainable ingredients and the circular use of by-products [55]. Fish-oil-based omega-3s, once thought to reduce CVD risk, are now questioned due to their limited efficacy and side effects [56]. Researchers are exploring polar lipids (PL) from marine sources as alternatives to reduce platelet aggregation and inflammation by inhibiting Platelet Activating Factor (PAF) [8,57]. Microalgae are also emerging as a promising source of high-value metabolites including polyphenols and other antioxidant compounds [58]. Nevertheless, further studies in microalgae culture conditions and nutraceutical extraction methods are needed to mass-produce them [59].

Olive pomace and other fruit pomaces, which are rich in anti-inflammatory compounds, can be used to improve food production processes and functional properties [60]. New supplements from polyphenol-rich sources such as grape pomace, berries, and pomegranates are being tested, though not all yield metabolic benefits [61]. Continued research and clinical trials are essential to confirm the effectiveness of functional supplements.

### 4.4. Food By-Products Used as Prebiotics

In recent decades, there has been a significant increase in scientific and technological interest in the valorization of agroindustrial by-products, with the aim of reducing food waste and transforming these materials into viable sources of bioactive compounds.

One of the most innovative and sustainable approaches in this context is the exploration of the prebiotic potential of such by-products, which often contain relevant amounts of non-digestible carbohydrates, fermentable oligosaccharides, and phenolic compounds, all capable of modulating the gut microbiota [62]. According to the updated definition by Gibson et al. [63], prebiotics are substrates selectively utilized by host microorganisms that confer health benefits. These include improving digestive function, strengthening the immune system, and enhancing the production of bioactive metabolites such as short-chain fatty acids (SCFAs), which are essential for gut health.

Among the most widely studied types of prebiotics are fructooligosaccharides (FOSs), found in vegetables such as garlic, onion, banana, asparagus, sugar beet, wheat, barley, and tomato; galactooligosaccharides (GOSs), derived from human and cow milk; inulin and its derivatives, mostly extracted from chicory; and xylooligosaccharides (XOSs), isomaltooligosaccharides, and maltooligosaccharides obtained from starch [64,65]. Other compounds such as lactulose, lactosucrose, and soybean oligosaccharides have also been recognized for their prebiotic functionality, meeting the key criteria of resistance to digestion in the upper gastrointestinal tract, fermentability by beneficial bacteria, and the promotion of the growth or activity of specific strains such as *Bifidobacterium* and *Lactobacillus* [66].

Several recent reviews on the valorization of fruit and vegetable processing by-products indicate that co-products from tropical fruits such as passion fruit, acerola, mango, orange, banana and jabuticaba are rich sources of dietary fiber and polyphenols with potential prebiotic activity, and that their incorporation into fermented dairy or plant-based beverages can enhance the growth and metabolic activity of probiotic lactic acid bacteria, increasing short-chain fatty acid and lactic acid production and, in some formulations, contributing to folate biosynthesis [67,68,69]. However, substrate selectivity is a key factor in classifying a compound as a prebiotic. Some materials, such as orange-derived by-products, have been shown to simultaneously promote the growth of pathogenic bacteria, thereby compromising their functional applicability [66].

In addition to their direct effects on gut microbiota, agro-industrial by-products with prebiotic activity may offer other potential benefits, such as enhancing intestinal barrier function, modulating immune responses, and improving probiotic adhesion to the intestinal mucosa, an essential aspect for colonization and persistence [69,70]. For instance, aqueous fruit extracts have been shown to increase *Lactobacillus rhamnosus* adhesion to intestinal epithelial cells in *in vitro* models, even under co-culture with competing strains. This effect may be associated with the presence of polyphenols and their antimicrobial action against pathogenic microorganisms, which creates a more favorable environment for probiotics.

Some studies reported increases of approximately 20–45% in probiotic adhesion to intestinal epithelial cells following supplementation with polyphenol-rich fruit extracts and prebiotic compounds derived from agro-industrial by-products.

The strategic combination of prebiotics and probiotics in synbiotic formulations has become a rapidly expanding area of research. Recent studies and reviews indicate that the incorporation of prebiotic substrates derived from agro-industrial by-products, such as apple peel, prickly pear peel or other fruit fibers, into alginate-based microcapsules significantly improves the viability, stability and gastrointestinal resistance of encapsulated probiotic strains, enhancing their survival during storage, as well as under simulated gastric and intestinal conditions [71]. Additionally, the inclusion of FOS improves the surface structure of the capsules by reducing porosity and increasing the protection of viable bacteria.

In the field of animal nutrition, the use of by-products with prebiotic potential has also gained relevance. For example, chicory, citrus pulp, and rye bran have been shown to improve intestinal integrity using *in vitro* models of piglet gastrointestinal tracts by positively regulating the expression of genes associated with tight junctions, more effectively than conventional inulin fermentation [72]. These findings suggest that some by-products could serve as functional alternatives to support gut health during weaning, a critical stage in animal production.

Despite progress in this area, there are still important methodological challenges, particularly regarding the standardization of in vitro fermentation protocols used to assess prebiotic activity. More studies are also needed to directly correlate *in vitro* results with observed *in vivo* effects, which would validate the functionality of these ingredients and support their inclusion in the development of functional foods. According to Health Canada’s definition, functional foods are those that, in addition to their nutritional value, provide demonstrate physiological benefits that contribute to the prevention of chronic diseases [73]. In this sense, agro-industrial by-products enriched in prebiotic compounds not only represent a promising strategy for waste valorization but also offer real opportunities for the innovative development of sustainable, accessible, and health-promoting functional foods. In summary, by-products represent a promising source of bioactive ingredients with potential benefits for metabolic health, inflammation, and gut function. However, most of the supporting evidence derives from *in vitro* and animal studies, while human trials remain limited in number and scope. To translate these findings into public health impact, further clinical validation, standardized safety assessments, and regulatory clarity are required. Addressing these gaps will determine whether valorized by-products can evolve from promising experimental applications into reliable components of sustainable functional foods. Furthermore, although some meta-analytical evidence supports the beneficial effects of specific bioactive compounds such as polyphenols and bioactive peptides, additional large-scale and standardized clinical investigations are still necessary to establish dose–response relationships, long-term safety, and regulatory applicability (Table 2).

## 5. Transformation Processes and Valorization Technologies

Processing technologies play a crucial role in transforming food by-products into valuable ingredients or food products. Techniques such as drying, extrusion, fermentation, enzymatic hydrolysis, hydrothermal treatments, chemical treatments, and nanotechnology are key in enhancing the functional properties and nutritional value of these by-products. Drying helps preserve the shelf life of the by-products, while extrusion processes enable the creation of new textures and forms in food products. Fermentation and enzymatic hydrolysis can further improve the digestibility and bioavailability of nutrients, creating functional ingredients with added health benefits. Hydrothermal treatments, which involve high-pressure or high-temperature processes, are effective in breaking down complex compounds and enhancing the extraction of bioactive molecules. Chemical treatments, on the other hand, are widely used to modify the chemical structure of by-products, increasing their potential as food ingredients. In recent years, nanotechnology has shown promise in enhancing the bioactivity and stability of bioactive compounds extracted from food by-products. Additionally, green technologies, which focus on environmentally friendly processes, are becoming increasingly important. These innovations not only increase the value of food by-products but also contribute to reducing waste and minimizing environmental impact, supporting the development of a more sustainable and circular food system [74,75,76].

Recent advances in food by-product valorization have shifted from predominantly conceptual sustainability approaches toward more quantitative and technology-oriented strategies. Emerging transformation technologies such as ultrasound-assisted extraction, microwave-assisted extraction, supercritical fluid extraction, enzymatic hydrolysis, membrane separation, and fermentation processes are increasingly being applied to improve extraction efficiency, reduce solvent consumption, minimize environmental impact, and enhance the recovery of high-value bioactive compounds for functional food applications.

Ultrasound-assisted extraction (UAE) has been reported to improve extraction yields by approximately 25–40% while reducing extraction time by nearly 50% compared to conventional solvent extraction methods [16]. Similarly, microwave-assisted extraction (MAE) may reduce solvent consumption by 30–40% and significantly enhance the recovery of thermolabile bioactive compounds. Supercritical fluid extraction (SFE), although associated with higher equipment costs, provides higher extract purity and substantially lower environmental impact due to reduced organic solvent use (Table 3).

### 5.1. Environmental Impact

The environmental impact of new food developments from by-products is significantly lower compared to conventional production models. These innovative approaches align with global sustainability goals by addressing critical issues such as waste reduction, carbon footprint mitigation, and efficient resource use [22,46]. The development of food from by-products represents a key strategy for improving the sustainability of the food industry and reducing the environmental impact associated with conventional production [77]. Furthermore, the use of by-products in food processing not only optimizes processes but also provides bioactive compounds that are beneficial for health, contributing to more sustainable practices in the industry [21,78]. Emerging technologies for resource recovery in the food industry show that by-product-based production has a considerably lower environmental impact than conventional production methods, reinforcing the need for innovative approaches in the food industry [9,79]. These contributions underline the importance of integrating by-product reuse into food production systems to optimize processes and contribute to a more sustainable future.

Several studies reported greenhouse gas emission reductions ranging from 20% to 30% following the implementation of by-product valorization strategies compared with conventional disposal practices. Additionally, reductions in water consumption and landfill-associated methane emissions have been reported.

### 5.2. Reduction in Resource Use

One of the most significant environmental benefits of utilizing food by-products lies in reducing the demand for primary resources. Traditional food production often relies on extensive land use, high water consumption, and energy-intensive processes. By repurposing by-products, the need for new raw materials is minimized, thereby conserving natural resources. For example, some reports highlighted that reusing by-products such as fruit peels, vegetable residues, and spent grains reduces the environmental burden associated with cultivating additional crops for similar functional ingredients. This shift contributes to lowering deforestation rates and mitigating water scarcity, particularly in regions with limited resources [80].

Economic analyses indicate that a substantial proportion of food processing by-products can be reutilized for high-value applications, reducing waste management costs while generating additional revenue streams through the production of functional ingredients, nutraceuticals, and bio-based products [9,22].

Additionally, by-products are often rich in nutrients and bioactive compounds, which can be harnessed without the environmental costs associated with conventional extraction methods. For instance, the recovery of protein and dietary fiber from spent grains eliminates the need for new agricultural production while providing valuable components for human nutrition [81,82].

### 5.3. Carbon Footprint Reduction

The production and disposal of food waste contribute significantly to greenhouse gas (GHG) emissions, including methane emissions from landfills and CO_2_ emissions from incineration. The valorization of by-products offers an effective solution to these issues. According to a study by Rao et al. [22], incorporating by-product valorization into the supply chain can reduce GHG emissions by up to 30%, depending on the scale of implementation and the type of by-product utilized.

For example, the transformation of dairy industry by-products such as whey into high-protein powders not only prevents waste but also reduces the carbon footprint of producing equivalent protein sources from fresh dairy inputs. Similarly, citrus peels, often discarded as waste, can be converted into valuable pectin and flavonoid extracts, reducing the need for energy-intensive synthetic alternatives [83].

Large-scale valorization strategies could prevent millions of tons of food waste annually while reducing landfill-associated methane emissions and improving resource efficiency throughout the food supply chain [84].

### 5.4. Aligning with Global Sustainability Goals

By integrating environmental and economic benefits, by-product valorization supports global policy initiatives such as the United Nations Sustainable Development Goals (SDGs), particularly Goal 12: Responsible Consumption and Production. This approach also aligns with the European Green Deal, which emphasizes the transition to a circular economy and reducing environmental impacts across all sectors [11].

In conclusion, the environmental impact of utilizing food by-products is transformative, reducing waste, conserving resources, and lowering GHG emissions. Coupled with economic benefits, these advancements underscore the potential of by-product valorization as a key strategy for achieving a sustainable food system.

The utilization of by-products in food production has been identified as a key component in achieving the sustainable development goals (SDGs). Authors such as Vilariño et al. [82] emphasize that by-product valorization not only contributes to reducing food waste but also promotes responsible consumption and production practices, aligning with SDG 12. This goal aims to ensure sustainable consumption and production patterns, which is critical in a context where resource scarcity and climate change pose significant global challenges.

Emerging technologies for resource recovery in the food industry show that by-product-based production can have a considerably lower environmental impact than conventional methods, reinforcing the need for innovative approaches in the food industry. Reviews indicate that integrating agri-food by-products into production systems supports environmental sustainability through waste minimization, resource efficiency, and value creation from residual streams [85,86]. These contributions underline the importance of embedding circular economy principles into food production to optimize processes and contribute to a more sustainable future.

Several studies reported greenhouse gas emission reductions ranging from 20% to 30% following the implementation of by-product valorization strategies compared with conventional disposal practices.

Additionally, recent studies indicate that the efficient use and valorization of food by-products can contribute significantly to climate change mitigation. Research grounded in circular economy frameworks shows that optimizing resource use and reducing waste across food production and distribution systems can lower greenhouse gas emissions and improve energy efficiency, thereby reducing the overall environmental footprint of the food sector [11,87]. This integrated approach supports both SDG 12 on responsible consumption and production and SDG 13 on climate action, highlighting the need for coordinated measures to address climate change within food systems.

In summary, the comparison with other studies underscores the critical role of utilizing by-products in food production as an effective strategy for advancing a more sustainable and responsible food system. The accumulated evidence suggests that this practice is not only environmentally beneficial but also provides significant economic opportunities for producers.

## 6. Standardization of Processes

A major hurdle in the valorization of food by-products lies in the variability of their composition, which is influenced by factors such as raw material origin, seasonal fluctuations, and processing methods. This variability often results in inconsistent product outcomes, hindering their integration into standardized food production systems [88]. Establishing industry-wide guidelines and standards is critical for ensuring safety, quality, and functionality. Collaborative efforts among regulatory agencies, research institutions, and industry stakeholders can lead to the development of robust frameworks for the collection, processing, and certification of by-products. Technologies including blockchain can be leveraged to enhance traceability and transparency in the supply chain, ensuring that by-products meet established standards and gain consumer trust [89,90,91,92,93].

### Consumer Acceptance

Consumer perception represents a major challenge to the large-scale adoption of by-product-derived foods, as consumers often associate terms such as “waste” or “by-products” with inferior quality or safety concerns. Addressing these barriers requires a dual strategy, combining improvements in sensory quality with reshaping public perception through education and transparent communication. Campaigns that emphasize the environmental, nutritional and functional value of foods derived from upcycled by-products have been identified as an important enabling factor for consumer acceptance. In this context, reviews on upcycling technologies highlight that transparent communication and positive framing are essential to overcoming negative associations with food by-products and to support market uptake [86]. Labels and positive framing, such as emphasizing sustainability and frugality, have shown promise in improving consumer acceptance and the perceived value of upcycled foods, especially when environmental and nutritional information is highlighted [87,88,89]. Moreover, actively involving consumers in sustainability narratives, such as showcasing the reduction in carbon footprint or waste achieved through their purchase choices, can foster stronger emotional engagement and long-term consumer loyalty.

## 7. Economic Viability

The environmental benefits associated with the utilization of food industry by-products are intrinsically linked to substantial economic advantages. The economic cost of food loss and waste impact on the global economy is estimated at approximately USD 1 trillion annually, representing an enormous reservoir of recoverable value [91]. Reducing waste management costs, such as landfill fees and incineration, translates into immediate financial savings for food producers. A particularly illustrative case is that of brewers’ spent grain (BSG), the main solid by-product of the brewing industry, which represents approximately 85% of total solid residues. When it is not valorized, breweries must dispose of it at a cost of up to EUR 100 per ton in the EU, while its current market value as cattle feed is as low as EUR 35/ton; furthermore, landfilling each ton of BSG releases the equivalent of 513 kg of CO_2_, adding environmental costs to the economic burden [92,93]. These figures make a compelling economic case for valorization: redirecting the same material toward high-value functional food ingredients eliminates disposal costs and generates positive revenue simultaneously.

Moreover, transforming by-products into high-value products such as functional foods, nutraceuticals or bio-based materials opens up new revenue streams, as demonstrated by the whey industry. The global whey protein market size was USD 13.58 billion in 2025 and is projected to grow to USD 26.24 billion by 2034, exhibiting a CAGR of 7.62% during the forecast period. This growth is driven by demand for sports nutrition and functional foods, such as infant formula [94,95]. This added value stands in high contrast to the disposal costs dairy producers once faced when this stream was treated merely as industrial effluent. The transition from a waste liability to a multi-billion-dollar ingredient market perfectly exemplifies the economic logic of by-product valorization.

Studies on circular economy implementation indicate that companies adopting circular strategies often enhance both environmental and economic performance by minimizing waste, reducing dependence on virgin raw materials and increasing resilience to price volatility in agricultural markets [14,96]. From a systemic perspective, circular business models have also been associated with lower GHG emissions and improved energy and material efficiency, contributing to the achievement of SDG 12 (responsible consumption and production) and SDG 13 (climate action) [14,97]. However, economic viability remains a challenge, especially for small and medium enterprises (SMEs), due to high initial investments in advanced processing technologies. Governmental support in the form of incentives, subsidies, or low-interest loans can be critical in overcoming these barriers. At the European level, for example, the Farm to Fork Strategy (European Commission, 2020)—one of the pillars of the EU Green Deal—explicitly includes support measures for SME food processors, small retail and food service operators through tailored solutions to promote new skills and business models, financed through instruments such Horizon Europe, the European Regional Development Fund (ERDF), and the European Agricultural Fund for Rural Development (EAFRD). Complementarily, the Circular Bioeconomy Partnership under Horizon Europe, established by Council Regulation (EU) 2021/2085 [98], explicitly targets the sustainable valorization of biological waste from agricultural and industrial sectors through multiscale biorefinery approaches, aiming to expand the circular economy at a regional and industrial scale.

Additionally, innovative business models, such as resource-sharing networks or cross-sector partnerships, can reduce costs and drive value creation. As noted above, collaborations between breweries and bakeries to reuse spent grains, or between dairy and nutraceutical industries to repurpose whey, illustrate the potential of synergistic approaches. Public/private initiatives such as the Waste4soil program (funded under Horizon Europe) further validate this model of converting food processing waste into bio/based fertilizers and soil improvers, with an ambitious goal of reducing chemical fertilizer dependence by up to 80% and generating direct economic benefits for local food industries through lower waste management costs and access to cheaper renewable inputs. Demonstrating the long-term financial benefits of these initiatives is essential for encouraging widespread adoption and scaling sustainable innovation in the food sector [99,100].

## 8. Future Perspectives

Future perspectives in food by-product valorization will likely be driven by the integration of artificial intelligence, machine learning, precision fermentation, metabolomics, and advanced green extraction technologies aimed at improving process optimization, scalability, and sustainability. AI-assisted predictive modeling and digital process simulation are increasingly being explored to optimize extraction yields, reduce solvent consumption, and improve the industrial reproducibility of bioactive compounds derived from food processing residues.

Technological advancements are at the heart of overcoming challenges in by-product utilization. Emerging processing methods such as enzymatic hydrolysis, supercritical fluid extraction, and advanced fermentation are transforming waste streams into high-value products [101]. These technologies enable the recovery of bioactive compounds, dietary fibers, and proteins, while preserving their functional integrity. Research into bioconversion processes, including the use of microbial and enzymatic systems, holds promise for converting low-value by-products into high-value food ingredients [102]. The adoption of these technologies requires strategic investments in research and development (R&D), as well as partnerships between academia and industry to pilot scalable solutions. The translation from laboratory-scale findings to industrial implementation remains one of the most persistent bottlenecks: while extraction yields for compounds such as pectins from citrus peels or omega-3-rich oils from fish by-products have been optimized at bench scale, cost-effective scale-up requires further engineering innovation and techno-economic validation.

Collaboration across sectors is essential to unlocking the full potential of by-product valorization. Academic institutions can drive innovation through cutting-edge research, while industries can provide practical insights into market needs and operational constraints [103]. Governments can serve as catalysts by offering policy support, funding, and infrastructure development. International collaborations, particularly in regions with abundant food waste but limited technological capacity, can amplify the impact of by-product valorization efforts. Knowledge-sharing initiatives, capacity-building programs, and cross-border partnerships can ensure that technological advancements are accessible to diverse stakeholders [103,104].

In parallel, several international collaborative initiatives and funding programs are supporting the transition toward circular and sustainable food systems. In Europe, the Horizon Europe and Circular Bio-based Europe Joint Undertaking (CBE JU) programs are currently investing hundreds of millions of euros in projects related to food waste valorization, sustainable biorefineries, and bio-based functional ingredients. Similarly, the European Green Deal and Farm to Fork Strategy continue promoting industrial sustainability and reductions in food-chain waste through public/private collaborative frameworks [105].

Regional and local initiatives are also increasingly emerging in Latin America, Asia, and other developing regions, where agro-industrial residues represent an important environmental and economic challenge. These programs aim to promote sustainable extraction technologies, local bioeconomy development, and the valorization of agricultural and food-processing by-products through collaborations between universities, research centers, governments, and industrial sectors [106].

Future research should focus not only on improving extraction efficiency and technological scalability but also on addressing regulatory harmonization, consumer acceptance, clinical validation, life-cycle assessment (LCA), techno-economic analysis (TEA), and long-term environmental impacts associated with industrial implementation of food by-product valorization strategies.

A particularly transformative development for the field is the accelerating integration of artificial intelligence (AI) and machine learning (ML) into food by-product valorization research. AI addresses several of the most persistent bottlenecks simultaneously. In the context of extraction process optimization—one of the most resource-intensive steps in bioactive recovery—artificial neural networks (ANNs) and hybrid ANN-genetic algorithm models have demonstrated superior predictive accuracy compared to conventional response surface methodology (RSM), with R^2^ values exceeding 0.97, enabling the optimization of complex, non-linear extraction parameters (such as temperature, solvent concentration, ultrasound power, and time) with substantially fewer experimental runs [107]. These AI-driven approaches have been applied to predict polyphenol yields from almond skin by-products under microwave-assisted extraction, to optimize supercritical fluid extraction of pomegranate seed oil, and to model the ultrasound-assisted extraction of bioactives from fruit and vegetable residues, consistently outperforming traditional mathematical models in reproducibility and energy efficiency. Reviews of the AI-driven optimization of bioactive compound extraction from food by-products highlight that such models deliver higher yields, lower energy use, and improved process consistency, positioning AI as a key enabling technology for the scale-up challenge that currently limits industrial adoption [108].

Beyond extraction, AI is being applied across the entire valorization pipeline. In functional food formulation, machine learning models—including support vector machines, random forests, and deep learning architectures—are being used to predict the bioactive potential, antioxidant activity, and shelf stability of by-product-derived ingredients, reducing the need for lengthy *in vitro* screening cascades [109]. AI also shows promise in gut microbiome modeling: machine learning tools applied to metagenomic datasets can predict the prebiotic selectivity of fibers and fermentable oligosaccharides derived from agro-industrial residues, helping to determine a priori which by-product fractions are likely to promote beneficial microbiota shifts versus those that risk stimulating pathogenic strains [52]—a critical issue identified in this review for by-products such as orange-derived fractions. Furthermore, generative AI and reinforcement learning approaches are beginning to be used in precision fermentation, accelerating the design of microbial systems capable of converting low-value by-products into specific bioactive metabolites. In the domain of supply chain and waste management, computer vision systems combined with predictive analytics have demonstrated the ability to optimize real-time spoilage detection and inventory management, contributing to waste reduction across the food production chain.

At the institutional level, the convergence of AI and food systems has prompted coordinated international action. In 2025, the Fraunhofer Heinrich Hertz Institute (HHI) and the International Telecommunication Union (ITU), together with the FAO, WFP, and IFAD, launched the Global Initiative on AI for Food Systems (AI4FS) at the AI for Good Global Summit [110], explicitly aiming to use AI to increase productivity, strengthen resilience, and promote global food security. The European Commission has also invested EUR 220 million in large-scale AI testing facilities that include food and agricultural applications, with operational services from 2024. Likewise, the USDA’s AI Institute for Next-Generation Food Systems (AIFS), hosted at UC Davis, supports over 40 ongoing research projects applying AI to sustainable food production [111]. These institutional commitments reflect a growing recognition that AI is not merely a supplementary analytical tool but a structural enabler for the transition from descriptive by-product characterization to predictive, scalable valorization systems.

Despite these advances, important limitations remain in the integration of AI into by-product valorization. Most AI models in this domain are trained on small, heterogeneous datasets that lack standardization in terms of extraction conditions, raw material origin, and analytical methods—the same variability that undermines conventional research. The interpretability of complex deep learning models also poses challenges for regulatory acceptance, since food ingredient approval processes require transparent, mechanistically justified evidence. Furthermore, the deployment of AI-driven optimization tools is resource-intensive and currently beyond the reach of most SMEs, particularly in lower-income regions. Addressing these gaps will require open-access food by-product databases, federated learning frameworks that allow multi-institution model training without sharing proprietary data, and the development of explainable AI (XAI) approaches suited to food science applications. Ultimately, the combination of advanced green extraction technologies, robust clinical evidence, harmonized regulatory pathways, and AI-enabled process intelligence represents the most credible path toward transforming food industry by-products from underutilized residues into reliable, high-value components of sustainable food systems.

## 9. Conclusions

This review collects evidence from 2015 to 2026 demonstrating that food industry by-products represent a scientifically and technologically mature resource for the development of functional ingredients and sustainable food products. However, the path from laboratory discovery to market-ready application remains fragmented and uneven across sectors. Beyond the synthesis, certain points of conflict can be identified that deserve discussion.

First, a notable discrepancy exists between the abundance of *in vitro* and animal model findings and the scarcity of robust human clinical trials. Most of the reported health benefits (antioxidant activity, prebiotic activity, anti-hypertensive effects) are primarily supported by cell-based assays or rodent studies. Human trials (at least those identified in this review) usually involve small sample sizes, short-term interventions and heterogeneous endpoints, which make cross-study comparisons unreliable. This contrasts with the optimistic approach found in many primary publications, which frequently extrapolate *in vitro* values or improvements observed in animal biomarkers directly to predicted outcomes for human health without proper qualification.

Second, inconsistencies persist in the reported composition and functional yields of by-products across studies. These differences underscore the need for standardized protocols for raw material characterization, extraction, and biological activity assessment before such ingredients can be reliably incorporated into functional food formulations.

Third, the environmental narrative surrounding by-product valorization, while compelling, requires more nuanced quantification. For example, GHG emission report studies are highly dependent on context—varying substantially with geography, energy mix, transport logistics, and the specific valorization pathway chosen, which limits direct comparison. Future research should adopt harmonized LCA frameworks to allow cumulative and comparative environmental impact assessments across by-product categories and processing technologies.

Looking forward, the valorization of less-studied by-product streams—such as camel milk permeate or microalgal biomass residues—offers genuine innovation potential that the current literature has barely begun to explore. The integration of AI and machine learning into this field is no longer a distant prospect but an active and rapidly maturing reality: AI-powered models already outperform conventional methods in bioactive extraction optimization, machine learning tools are being applied to predict prebiotic selectivity and gut microbiome interactions, and international institutional frameworks such as the ITU-FAO Global Initiative on AI for Food Systems (launched 2025) signal a structural commitment to AI-enabled food system transformation. Nevertheless, the deployment of these tools must be accompanied by open-access standardized databases, explainable AI frameworks compatible with food regulatory requirements, and capacity-building efforts that make AI-driven optimization accessible beyond well-resourced research centers. In sum, the field has generated substantial and promising foundational evidence; the critical next step is translational rigor—moving from descriptive characterization toward well-designed clinical trials, standardized methodologies, and scalable, AI-supported, economically validated processes that can genuinely advance by-product valorization from laboratory innovation to public health impact.

## Figures and Tables

**Table 1:** Summary of scientific articles (2018 to present) that involve the development of foods or functional ingredients based on by-products.

Matrix	By-Product	Target Compound/Ingredient	Technology	Extraction Parameters	Yield (%)	Advantages	Limitations	Outcome/Application	Reference
Dairy	Whey	Galacto-oligosaccharides (GOS)	Enzymatic transgalactosylation	40–50 °C; β-galactosidase; 2–6 h	35–45%	High prebiotic potential	Enzyme cost	Prebiotic activity	[27]
Dairy	Whey	Bioactive peptides	Enzymatic hydrolysis (e.g., Alcalase)	pH 7–8; 50–55 °C	20–30%	High antioxidant activity	Peptide instability	Antioxidant; antihypertensive	[31]
Dairy	Fermented whey	Bioactive metabolites/peptides	Fermentation (kefir; LAB)	24–48 h; 30–37 °C	Variable	Improved bioavailability	Fermentation variability	Antimicrobial; immunomodulatory	[28,29]
Dairy	Colostrum	Lactoferrin; milk oligosaccharides	Microfiltration/isolation	Low-temperature filtration	High purity	Preserves bioactivity	High operational cost	Immunomodulatory; antioxidant	[26]
Vegetable/Fruit	Olive mill wastewater	Hydroxytyrosol	Membrane processes	Pressure-driven filtration	60–80% recovery	Reduced solvent use	Membrane fouling	Preservative; functional ingredient	[32]
Marine	Fish skins/bones/viscera	Collagen; peptides; oils	Hydrolysis	Enzymatic; 40–60 °C	20–50%	High-value biomolecules	Stability issues	Nutraceuticals; wound healing; functional foods	[33]
Marine	Shrimp shells/heads	Chitosan; astaxanthin; protein hydrolysates	Enzymatic/chemical extraction	Acid/alkaline pretreatment	15–40%	High commercial value	Chemical waste generation	Preservatives; cosmetics	[34,35,36]
Marine	Crab cooking effluents	Concentrates/flavor fractions	Concentration & recovery	Thermal concentration	Variable	Reuse of industrial effluents	Flavor variability	Natural food flavorings	[37]

LAB: Lactic acid bacteria.

**Table 2:** Comparative levels of scientific evidence associated with food-industry by-product-derived functional compounds.

By-Product	Main Compound	Main Reported Effect	In Vitro Evidence	In Vivo Evidence	Clinical/Meta-Analysis Evidence	Main Limitation
Grape pomace	Polyphenols	Antioxidant and anti-inflammatory activity	ROS reduction and antioxidant activity	Reduced inflammation and oxidative stress	Limited clinical evidence	Lack of large-scale randomized trials
Whey peptides	Bioactive peptides	Antihypertensive effects	ACE inhibition	Blood pressure reduction in animal models	Small human intervention studies	Limited cohort size
Citrus peel	Pectin/flavonoids	Prebiotic and gut modulation effects	Increased probiotic growth	Gut microbiota modulation	Few clinical studies available	Standardization issues
Shrimp by-products	Chitosan; astaxanthin	Anti-inflammatory and antioxidant effects	Cytoprotective activity	Improved metabolic parameters	Limited translational evidence	Extraction variability
Olive mill wastewater	Hydroxytyrosol	Antioxidant and cardioprotective effects	Radical scavenging activity	Reduced oxidative stress markers	Preliminary meta-analytical evidence	Limited long-term clinical validation

**Table 3:** Comparative analysis of emerging technologies applied to food industry by-product valorization.

Technology	Main Application	Yield Improvement	Solvent Reduction	Time Reduction	Main Advantages	Main Limitations	Industrial Scalability
Ultrasound-assisted extraction (UAE)	Recovery of polyphenols, antioxidants, proteins	+25–40%	30–50%	~50%	Fast extraction, lower energy consumption, preservation of thermolabile compounds	Difficult scale-up and equipment optimization	Moderate to high
Microwave-assisted extraction (MAE)	Polyphenols, carotenoids, bioactive compounds	+20–35%	~40%	50–60%	Rapid heating, reduced processing time, improved extraction efficiency	Possible thermal degradation and uneven heating	Moderate
Supercritical fluid extraction (SFE)	Lipids, carotenoids, essential oils	High purity extracts	Up to 80% reduction in organic solvents	Moderate	Green technology, solvent-free extracts, high selectivity	High operational and equipment costs	Moderate
Enzymatic hydrolysis	Protein and peptide recovery	Variable depending on substrate	Minimal solvent use	Moderate	Mild conditions, improved digestibility and bioactivity	Enzyme cost and process optimization	High
Fermentation technologies	Functional foods, probiotics, prebiotics	Improves bioavailability and functionality	Low solvent requirement	Long processing time	Sustainable, microbiota modulation, enhanced nutritional value	Microbial stability and contamination risks	High
Membrane separation technologies	Fractionation and purification of bioactive compounds	High selectivity	Reduced chemical usage	Moderate	Continuous processing, low thermal damage	Membrane fouling and maintenance costs	High
Hydrothermal and green technologies	Biomass pretreatment and compound recovery	Improved accessibility of bioactives	Reduced chemical consumption	Moderate	Eco-friendly processing, circular economy compatibility	Energy demand and operational optimization	Moderate to high

## Data Availability

No new data were created or analyzed in this study. Data sharing is not applicable to this article.

## Abbreviations

The following abbreviations are used in this manuscript:

AI	Artificial Intelligence
CVDs	Cardiovascular Diseases
FAO	Food and Agriculture Organization of the United Nations
FOS	Fructooligosaccharides
FOSHU	Foods for Specified Health Uses
GHG	Greenhouse Gas
GOS	Galacto-oligosaccharides
HHP	High Hydrostatic Pressure
LAB	Lactic Acid Bacteria
MAE	Microwave-assisted Extraction
ML	Machine Learning
NCDs	Non-communicable Diseases
PAF	Platelet Activating Factor
PL	Polar Lipids
PUFAs	Polyunsaturated Fatty Acids
R&D	Research and Development
SCFAs	Short-chain Fatty Acids
SDGs	Sustainable Development Goals
SFE	Supercritical Fluid Extraction
SHPH	Shrimp Head Protein Hydrolysates
SMEs	Small and Medium Enterprises
UAE	Ultrasound-assisted Extraction
USFDA	Food and Drug Administration of the United States
XOS	Xylooligosaccharides
